# Broken Suture Needle During Episiotomy Repair: Successful Localization and Removal Using Portable Radiography

**DOI:** 10.7759/cureus.106105

**Published:** 2026-03-30

**Authors:** Tugce Korhan, Ali Yavuzcan, Ozlem Moraloglu

**Affiliations:** 1 Department of Obstetrics and Gynecology, Ankara Bilkent City Hospital, University of Health Sciences, Ankara, TUR; 2 Department of Obstetrics and Gynecology, University of Health Sciences, Ankara, TUR

**Keywords:** broken suture needle, episiotomy, needle localization, portable radiography, postpartum complication, retained surgical foreign body

## Abstract

Episiotomy is a commonly performed surgical procedure during vaginal delivery and is generally considered safe. However, rare complications may occur during episiotomy repair, including fracture of the suture needle. Localization and retrieval of a broken needle fragment can be challenging, particularly in the presence of active bleeding or limited visualization of the surgical field.

We report a case of a fractured suture needle during episiotomy repair in a postpartum patient. Because the needle fragment could not be immediately located during the procedure, portable radiography was used to identify the exact location of the retained fragment. Following radiographic localization, targeted minimal dissection was performed, and the needle fragment was successfully removed within a short period of time. The patient remained hemodynamically stable throughout the procedure. No significant bleeding or postoperative complications were observed. Follow-up examination showed normal healing of the episiotomy site. This case highlights the usefulness of portable radiography as a practical and accessible method for the localization and safe removal of retained surgical needle fragments during episiotomy repair. Prompt identification and minimally invasive retrieval can prevent prolonged surgical exploration and potential complications.

## Introduction

Episiotomy is a commonly performed surgical procedure during vaginal delivery and is generally considered a minor intervention. However, as with all surgical procedures, rare but potentially serious complications may occur [1]. One of these complications is breakage or retention of a suture needle within the tissue during episiotomy repair.

Due to the rich vascularization and complex anatomical structure of the perineal region, localization of a retained needle can be particularly challenging, especially in the presence of active bleeding. Prolonged tissue exploration may increase blood loss and contribute to postpartum hemorrhage [2]. Conversely, leaving a foreign body in situ may lead to both medical and medicolegal complications, including chronic pain, dyspareunia, infection, abscess, or fistula formation [2].

Imaging plays a crucial role in the timely localization and safe removal of retained foreign bodies. Various modalities such as ultrasound, fluoroscopy, and radiography may be used depending on availability and clinical conditions. In this report, we present a case in which a suture needle broke during episiotomy repair and was lost within the perineal muscles, which was successfully localized and removed using portable radiography.

## Case presentation

A 25-year-old primigravid woman at 37 weeks and three days of gestation presented with rupture of membranes. The first stage of labor progressed uneventfully, and vaginal delivery was performed at 15:40 with a mediolateral episiotomy. Following placental delivery, episiotomy repair was initiated using 1/0 Vicryl with a 40-mm needle. During suturing, the distal one-third of the needle fractured, and the proximal portion remained within the tissue. Careful exploration of the perineal region failed to identify the needle either visually or by palpation.

**Figure 1:**
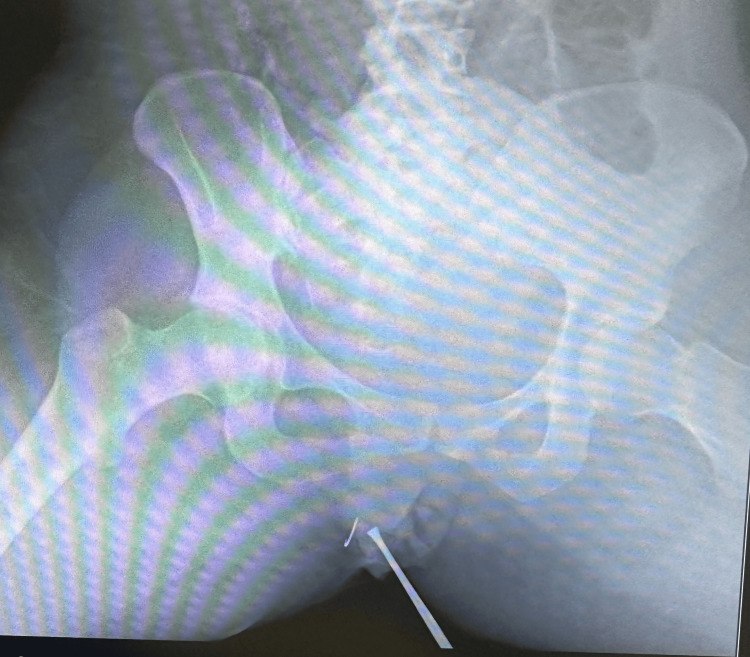
Radiographic localization with surface marking. The suspected target area was initially marked on the skin surface using a surgical instrument.

After administering additional local anesthesia, the previously sutured episiotomy line was reopened. However, despite repeated attempts, the fractured needle could not be located. As the patient was hemodynamically stable and there was no active bleeding, localization with portable radiography was planned. Portable anteroposterior (AP) pelvic radiography demonstrated a radiopaque foreign body in the right ischiorectal fossa (Figure 1).

**Figure 2:**
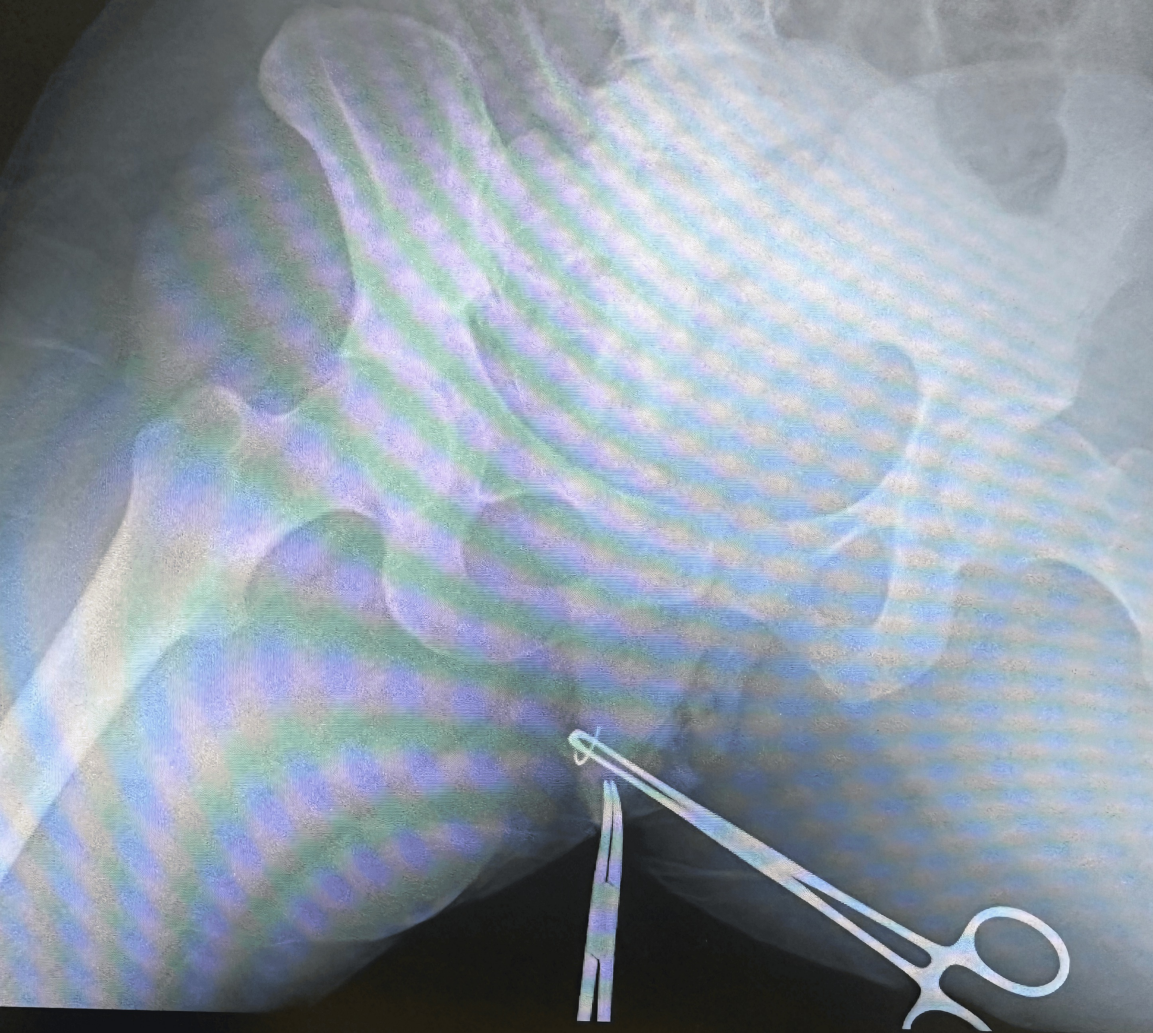
Intraoperative radiography-guided localization using metallic instruments. Intraoperative radiography-guided dissection was performed using a second metallic instrument positioned near the suspected target area to facilitate accurate localization of the retained needle fragment.

**Figure 3:**
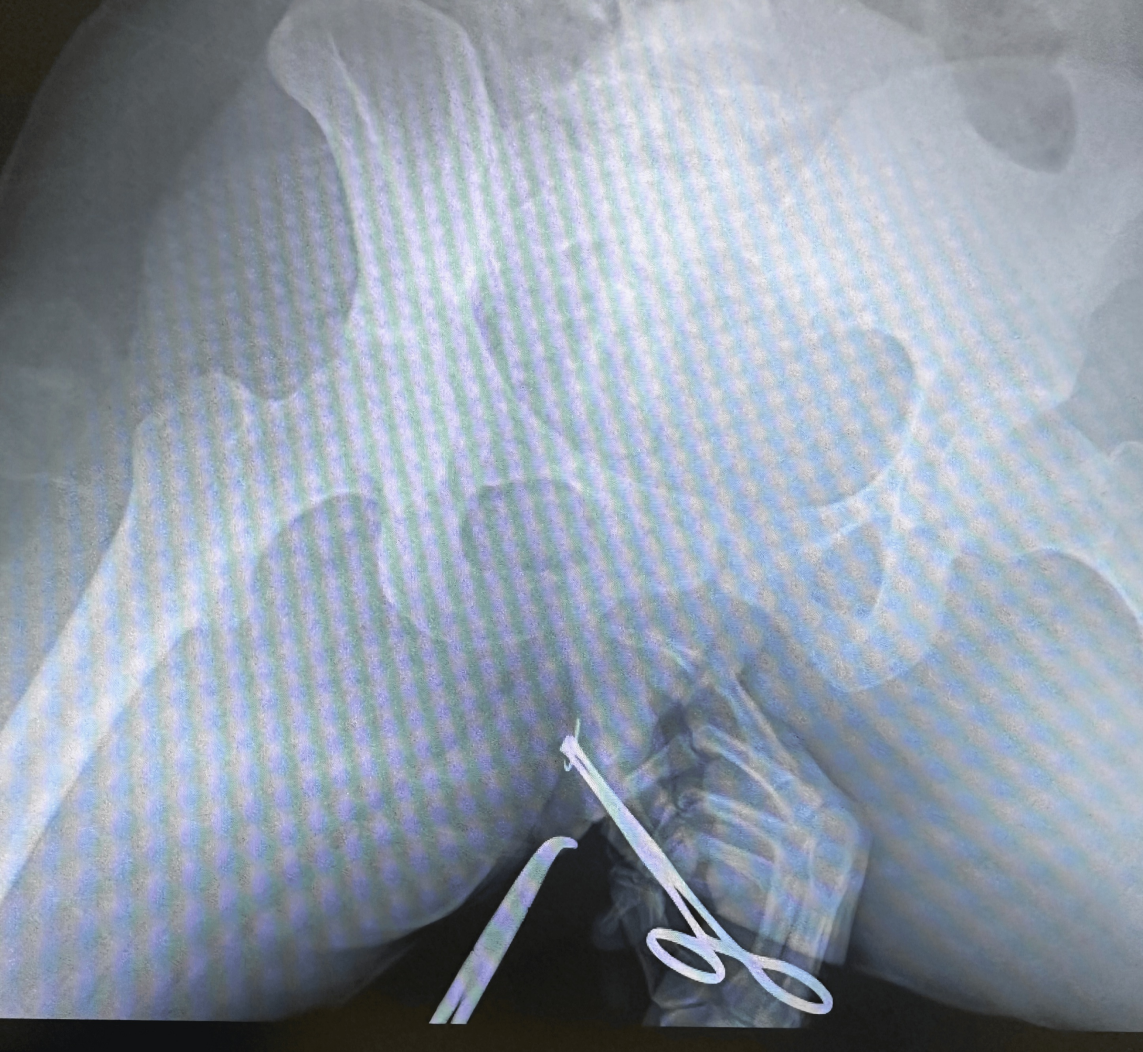
Radiographic-guided dissection of the needle fragment. Under real-time portable radiographic guidance, dissection was carefully performed toward the confirmed target area to expose the retained needle fragment prior to removal.

**Figure 4:**
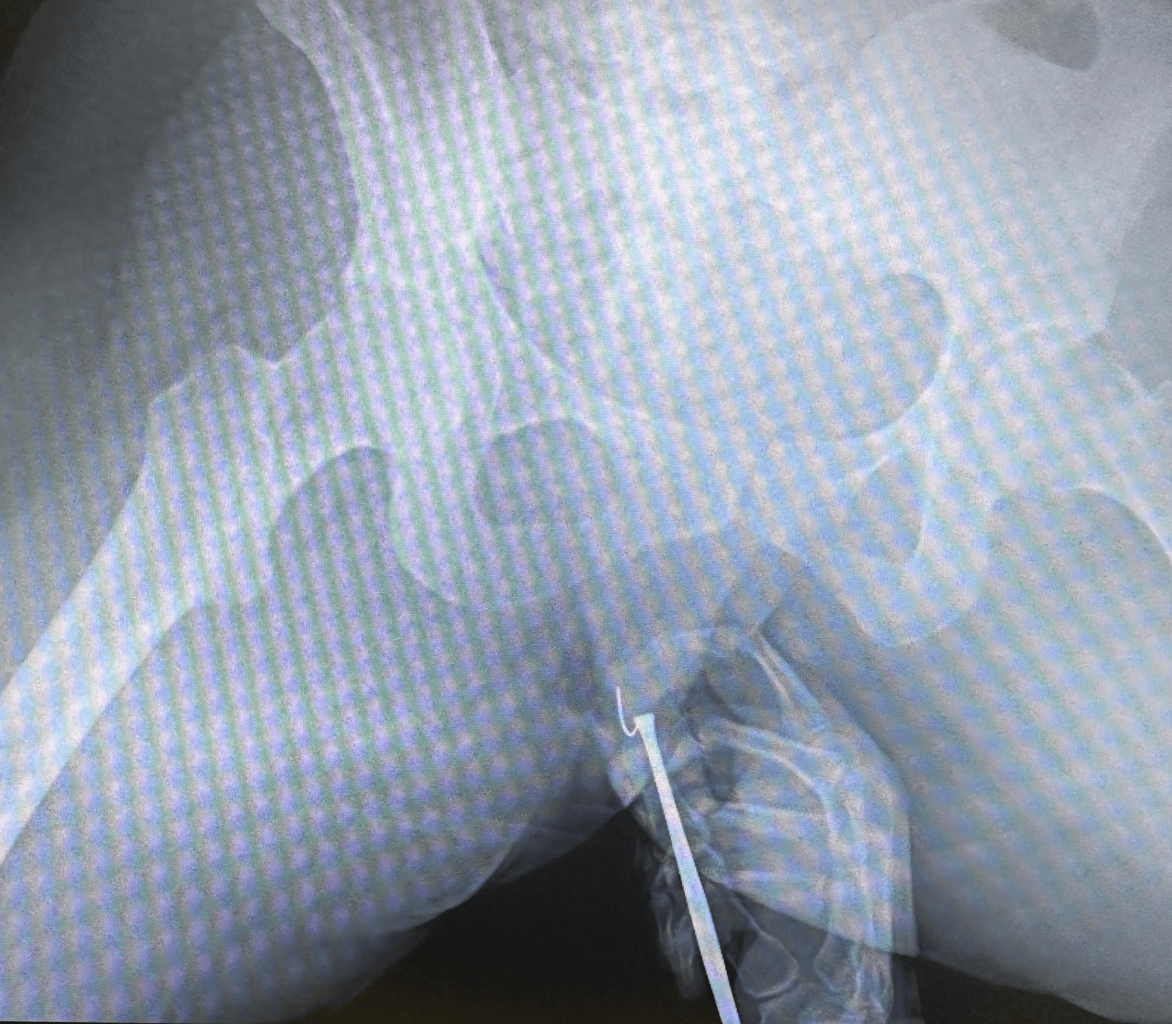
Successful retrieval of the fractured needle fragment. Successful retrieval of the fractured needle fragment following radiographic-guided localization and dissection.

Serial radiographic imaging combined with surface marking enabled accurate localization of the needle fragment (Figure 2). Intraoperative localization was further guided using a metallic probe under radiographic imaging (Figure 3). Under real-time portable radiographic guidance, targeted dissection was performed toward the confirmed location of the needle fragment. The fragment was successfully retrieved in approximately 20 min using minimal dissection with surgical instruments, including retractors and clamps (Figure 4).

Hemoglobin levels were 13.7 g/dL preprocedure, 13.5 g/dL at the second postpartum hour, and 11.5 g/dL at the sixth postpartum hour. No clinically significant bleeding was observed. Prophylactic cefazolin (1 g) was administered, and broad-spectrum antibiotics were continued until the seventh postpartum day. The episiotomy was anatomically resutured. Ultrasonographic evaluation at the second postpartum hour revealed no evidence of hematoma. The patient was mobilized and transferred to the ward in stable condition. Written informed consent was obtained from the patient for publication.

## Discussion

Needle breakage during episiotomy repair is a rare but stressful complication in the postpartum period. The incidence of this condition has been reported to be approximately 0.17%, although the number of published cases remains limited [3]. In such situations, the clinician must carefully weigh two potential risks. The first is bleeding and tissue trauma resulting from prolonged blind exploration performed in an attempt to locate the lost needle. Due to the highly vascular nature of the perineal region, prolonged surgical manipulation may increase blood loss and negatively affect wound healing. The second risk involves leaving the needle within the tissue, which may lead to long-term complications such as chronic pain, dyspareunia, infection, abscess formation, or fistula development [4,5].

In the present case, the patient’s hemodynamic stability allowed for controlled intervention under imaging guidance. Various imaging modalities have been described for the localization of retained foreign bodies, including ultrasound, fluoroscopy, and radiography. Ultrasound offers real-time imaging without radiation exposure; however, it is operator-dependent and may be less effective for detecting small metallic objects. Fluoroscopy (C-arm) provides dynamic intraoperative guidance but requires specialized equipment and may not always be readily available in delivery settings. However, C-arm fluoroscopy is widely used in other surgical specialties, particularly in general surgery and urology, for the intraoperative localization of foreign bodies [6]. In contrast, portable radiography is widely accessible, rapid, and effective in detecting metallic foreign bodies, making it a practical and reliable option in emergency settings. Because metallic needle fragments are radiopaque, portable radiography provided an accessible and sufficiently accurate alternative in this case. Targeted minimal dissection guided by imaging prevented unnecessary tissue trauma and contributed to a favorable postoperative outcome with uncomplicated postpartum recovery.

## Conclusions

Although episiotomy is generally considered a simple procedure, it is not completely risk-free. Performing episiotomy in unnecessary situations may expose patients to unexpected complications. Needle breakage during episiotomy repair is rare but requires careful management.

Localization using portable radiography may provide a safe and effective approach in hemodynamically stable patients. In cases where imaging is unavailable, referral to appropriate centers should be considered. Transparent management of the process and proper patient counseling are essential from both clinical and medicolegal perspectives.
